# The six-year outcome of alcohol use disorders in men: A population based study from India

**DOI:** 10.1016/j.drugalcdep.2016.02.039

**Published:** 2016-05-01

**Authors:** Abhijit Nadkarni, Helen A. Weiss, Aresh Naik, Bhargav Bhat, Vikram Patel

**Affiliations:** a Sangath, Goa, India; b London School of Hygiene & Tropical Medicine, London, UK; c Centre for Chronic Conditions and Injuries, Public Health Foundation of India, New Delhi, India

**Keywords:** Alcohol use disorders, Cohort, India

## Abstract

**Background:**

Despite the large and growing public health problem of alcohol use disorders (AUD) in India there is a dearth of evidence about the longitudinal outcomes in AUD. The aim of this study is to describe the course and outcomes of AUD in a population based sample of men in India.

**Methods:**

A community cohort of 1899 adult (18–49 years at baseline) men who participated in a cross-sectional survey in Goa, India between 2006 and 08, were re-interviewed face to face 6 years later (2012–14). A range of outcomes including social problems (e.g., workplace problems, domestic violence), morbidity (e.g., range of physical and mental health problems), biological parameters (e.g., mean corpuscular volume [MCV], gamma-glutamyl transpeptidase [GGT]) and mortality were measured at follow up. For the association of AUD at baseline with outcomes at follow-up, multivariable logistic regression was used to estimate odds ratios (OR). Analyses were weighted to account for baseline sampling design, age distribution, rural and urban sample sizes, number of adults aged 18–49 years in the household (at baseline), and non-response (at baseline).

**Results:**

1514 (79.7%) were seen at follow-up; a loss to follow up of 20.3%. At follow up, 3.7% of baseline non-drinkers and 15.0% of baseline casual drinkers had AUD. 46.9% of baseline hazardous drinkers and 55.4% baseline harmful drinkers continued to have AUD at follow up. Of those with AUD at baseline, 21.8% had stopped drinking at follow-up. Compared to being abstinent, harmful drinking at baseline was associated with several outcomes at follow-up: workplace/social problems, hypertension, death, tobacco use, suicidality, anxiety disorders, and raised GGT (p < 0.002). Hazardous drinking at baseline was associated with tobacco use and raised GGT and MCV (p < 0.002) at follow-up.

**Conclusion:**

Our findings of high persistent and new AUD in the community and the association with a range of long term adverse events are an important addition to the limited evidence about the course and outcomes of AUD in India, which have the potential for informing health policy.

## Highlights

• First community cohort study about longitudinal course of AUD from India.
• Over a short period of time, a substantial number of non/casual drinkers develop AUD.
• AUD lead to several long-term biopsychosocial adverse impacts in a LMIC like India.

## 1 Introduction

Alcohol Use Disorders (AUD) comprise a range of heterogeneous conditions related to excessive alcohol consumption and is recognised by the World Health Organization (WHO) as a distinct disorder; with hazardous drinking, harmful drinking and dependent drinking reflecting progressively more serious forms of the condition (Reid et al., 1999; WHO, 1994). AUD account for about 10% of Disability Adjusted Life Years (DALYs) caused by mental and substance use disorders, and an overwhelming majority (2.7 million) of the estimated 2.9 million deaths globally due to substance use disorders, are due to alcohol (Lim et al., 2012). In India, the prevalence of AUD among those who drink is relatively high (Prasad, 2009). The overall epidemiological picture of alcohol use in India is that almost half of all drinkers drink hazardously, and the signature pattern of hazardous drinking is one of heavy drinking, daily or almost daily drinking, under-socialized, solitary drinking of mainly spirits, drinking to intoxication and expectancies of drink-related dis-inhibition (Benegal, 2005). This results in high rates of alcohol-attributable mortality and prevalence of AUD relative to the per capita volume of alcohol consumed (Rehm et al., 2009).

Despite this large and growing public health problem, India does not have a national alcohol policy. One of the reasons for this is the lack of high quality contextual evidence about the problem. One type of evidence that helps to direct alcohol policy is the long-term course and outcomes in AUD. These have been studied extensively in developed countries (Finney et al., 1991, Gerdner and Berglund, 1997, Gual et al., 1999, Hyman, 1976, O’Connor and Daly, 1985), and find that AUD leads to higher mortality, morbidity and consequent health service utilization (Hyman, 1976, McCabe, 1986, O’Connor and Daly, 1985). More specifically, such studies have demonstrated associations of AUD with heart problems, sleeping difficulties, amnesic episodes, peptic ulcers, tuberculosis, liver disease, cerebro-vascular accidents, cerebellar ataxia, peripheral neuropathy, accidents, occupational problems, marital issues, financial difficulties and criminal convictions (McCabe, 1986, O’Connor and Daly, 1985). Finally, relapse and remission figures reported in patients with AUD vary. Mann et al. (2005) found 40% of their AUD patients to be abstinent while McCabe reported (1986) that 34.5% of an AUD cohort had become abstinent or controlled drinkers over the 16 year follow-up period, and 22% were experiencing continuing alcohol-related problems. Overall, recovery rates over various follow-up periods ranged between 14 and 40% (Gual et al., 1999, Mann et al., 2005, McCabe, 1986).

In India, longitudinal evidence of the course and outcomes of AUD is limited by small sample sizes, short follow-up periods and restriction to treatment seeking participants (Kar et al., 2003, Kuruvilla and Jacob, 2007, Kuruvilla et al., 2004, Mohan et al., 2002a, Mohan et al., 2002b, Singh et al., 2008), the latter being extremely prone to selection bias due to low help-seeking behaviours of men with AUD (Kohn et al., 2004). Further, as most AUD patients who are in contact with services do not have their AUD recognized, or receive evidence-based treatments, the effective treatment gap is likely to be even larger (De Silva et al., 2014). Hence it is important to understand the longitudinal history and outcomes of the majority of people with AUD in the community who do not get any treatment at all.

The aim of this study is to describe the longitudinal course of AUD in a population based sample of men. Our hypotheses are that in a community sample of men with AUD at baseline there is a high persistence of AUD and high prevalence of a range of adverse health (and associated biological parameters), and social outcomes at six years follow up. This is the first community-based cohort study of the course of AUD in India.

## 2 Material and methods

### 2.1 Setting

The study was conducted in Goa, which has a population of just over 1.4 million people, of whom 62% live in urban areas (Government of India, 2011). Unlike most of India, Goa has a more liberal culture towards drinking, reflected in lower abstinence rates. In Goa, the prevalence of current drinking in men was 39% in a community sample (Pillai et al., 2013), 59% in primary care (D’Costa et al., 2007) and 69% in industrial workers (Silva et al., 2003). Previous studies in Goa have reported the prevalence of hazardous drinking in men to be 15% in primary care (D’Costa et al., 2007) and 21% in an industrial male worker population (Silva et al., 2003).

### 2.2 Study design

In 2006–08, a cross-sectional survey (adults aged 18–49 years) was conducted in the following study sites: urban (beach areas popular among tourists and a typical commercial and residential area), and rural areas (six contiguous villages) of Northern Goa (Pillai et al., 2013). The villages were selected based on accessibility and population size required for the baseline study, as many villages in Goa are sparsely populated and some are remotely located mining areas. As is typical of this part of rural Goa, all these villages are socio-demographically homogeneous, and primarily depend on agriculture and seasonal brewing of Feni (the local alcoholic brew) during summer. A two stage probability sampling procedure, based on electoral rolls, was used to select the population based sample. From a randomly selected household the participants were selected at random from those of eligible ages within the households. Refusal rates for randomly selected households were 1.5%.

The study was designed as a retrospective community cohort study, comprising the 1899 men (only men were selected because of the low prevalence of drinking in Indian women) who were screened in the baseline survey and we measured a range of outcomes in the cohort at follow-up from September, 2012 to September, 2014.

### 2.3 Exposure

The principal exposure is AUD as detected during the baseline survey, defined by the 10 item Alcohol Use Disorders Identification Test (AUDIT) (Saunders et al., 1993). AUD was diagnosed using an AUDIT cutoff score of ≥8 and hence included hazardous, harmful and dependent drinkers (Saunders et al., 1993). The AUDIT has been validated in India (Pal et al., 2004), and used in cross-national studies, including India (Babu and Kar, 2010). For a previous study, the AUDIT has been translated into Konkani (Goan vernacular), using a systematic translation-back translation method with two teams of translators, followed by an item-by item analysis and selection by consensus (Silva et al., 2003). The cohort was made up of a range of exposures viz AUD (hazardous, harmful, dependent drinking), and casual drinking, and internal controls (i.e., abstainers).

### 2.4 Other baseline data

Baseline socio-demographic data were collected. Standard of Living Index (SLI) was computed as a wealth index and derived from information on ownership of household assets (Gwatkin et al., 2007). The SLI score was categorised as the lowest 40% (poor), middle 40% and highest 20% (rich). Asset-based indices have been found to be associated with consumption; and with development and health indices in India (Filmer and Pritchett, 2001).

### 2.5 Follow up procedures

All consenting participants were administered the self-report questionnaire by trained research workers. Standard protocolised procedures were adopted to measure height, weight and blood pressure, and for drawing and transporting blood samples. The research workers were blind to the exposure status of the participants to avoid misclassification of outcomes. Quality control was conducted by re-interviewing randomly selected participants by the research coordinator, random visits by the research coordinator to directly observe the research workers, and re-testing of randomly selected blood samples at an independent laboratory.

### 2.6 Follow up data

Besides the AUDIT score the following data was collected at outcome assessment:

#### 2.6.1 Self report using structured questionnaire

1. Problems at work directly related to drinking: These included four questions from the baseline survey which asked about any illness connected with drinking which kept the drinker from working on his regular activities for a week or more, losing or nearly losing a job because of drinking, people at work indicating that the drinker should cut down on drinking, and drinking hurting the chances for promotion, or salary increases or bonuses, or better jobs.
2. Number of work days lost due to poor health in past 28 days measured using an item derived from the WHO Health and Work Performance Questionnaire (HPQ; Kessler et al., 2003).
3. Marital problems related to drinking: These included two questions from the baseline survey which asked about a spouse getting angry with the participant about his drinking or the way the participant behaved while drinking, or a spouse threatening to leave the participant because of his drinking.
4. Questions from baseline survey about physical (slapped, hit, kicked, punched wife/partner or done something else that did or could have hurt her physically) and/or sexual abuse (had sex with wife/partner when he/she was unwilling or force him/her to do sexual things or to have sex) of partner/spouse.
5. Social problems: These included questions from the baseline survey about getting into a heated argument while drinking, getting into a fight while drinking, prominent people from society (e.g., community elder) questioning or warning the drinker because of his drinking, drinking contributing to the drinker hurting or harassing someone else emotionally, physically or sexually, getting into trouble because of drunk driving, and being caught/fined/threatened by the police or arrested for drunk driving.
  The questions about work, social, and marital problems are commonly used to assess social harm of drinking in the National Alcohol Surveys conducted by the Alcohol Research Group at Berkeley (Klingemann and Gmel, 2001).
6. Physical health problems measured using questions from the 10/66 Dementia Research Group population-based research programme for which one of the sites was India (Prince et al., 2007): Hypertension, heart disease, cerebrovascular accident (CVA) or Transient Ischaemic Attack (TIA), head injury with loss of consciousness, diabetes, COPD, and tuberculosis (TB).
7. Accidents or injuries.
8. Death: The cause of death was determined using the official death certificate.
9. Mental, Neurological and Substance Use (MNS) disorders
  (a) Current use of tobacco (smoked and/or chewed): Type (smoked, chewed etc), quantity, and frequency in past 12 months.
  (b) MNS disorders diagnosed using the Mini International Neuropsychiatric Interview (MINI 6.0) a validated short, structured diagnostic interview for DSM-IV and ICD-10 psychiatric disorders (Sheehan et al., 1998) used extensively in India (Salve et al., 2012).
  (c) Common Mental Disorders (CMD) assessed using the validated 12 item General Health Questionnaire (GHQ 12) (Goldberg, 1978) which has been widely used in the study setting (Patel et al., 1998, Patel et al., 2008).
10. Health service utilisation was measured using the adapted version of the validated Client Service Receipt Inventory (CSRI) (Chisholm et al., 2000), which has been used in the study setting (Patel et al., 2003).

#### 2.6.2 Clinical and biological outcomes

Blood pressure (BP), height, weight, Mean Corpuscular Volume (MCV) and Gamma Glutamyl Transferase (GGT). A MCV value of >92 fL and GGT value of >50 IU/L were coded as abnormal. A BMI of <18.5 kg/m^2^ or >24.9 kg/m^2^ was coded as positive for ‘unhealthy BMI’.

### 2.7 Ethics

Ethical approval was obtained from the Sangath Institutional Review Board (IRB), ethics committee of the London School of Hygiene and Tropical Medicine (LSHTM) and the Indian Council of Medical Research. Each research worker completed the NIH Protecting Human Research Participant online course. The results of the blood test and its interpretation were fed back to the participants. Participants with abnormal health parameters were offered referral to the local primary healthcare centre. Participants diagnosed with AUD or CMD were offered further free clinical assessment and treatment with by a psychiatrist.

### 2.8 Analyses

Baseline socio-demographic characteristics were described for the full cohort, and were compared between those who had and did not have AUD at baseline using chi square or one way ANOVA as appropriate. Baseline socio-demographic characteristics and baseline AUD status were compared between those who completed follow-up assessments and those who were lost to follow-up (LTFU). Multivariable logistic regression was used to identify factors independently associated with LTFU. For each exposure group at baseline (non-drinkers, casual drinkers, hazardous drinkers, harmful drinkers, AUD), the proportion followed-up was estimated, with the 95%CI.

The primary exposure of interest, baseline alcohol use was a categorical variable (abstainers, casual drinkers, hazardous drinkers, harmful drinkers), and all outcomes were binary variables. The abstainers and casual drinkers were not collapsed into a single category as they were significantly (p < 0.05) different with regard to area of residence, religion, employment status and SLI. For the association of AUD at baseline with outcomes at follow-up, logistic regression was used to estimate odds ratios (OR) and 95% confidence intervals (CI). This was done for each outcome separately. All outcome variables which were associated with baseline AUD at p < 0.1 on univariable analyses were fitted in separate models with baseline AUD adjusted for socio-demographic factors (age, SLI, marital status, educational status and employment status) using multiple logistic regression. The likelihood ratio test was used to estimate p-values for trend. Weights were applied to the data to account for the baseline sampling design, age distribution, rural and urban sample sizes, number of adults aged 18–49 years in the household (at baseline), and non-response (at baseline). To account for the multiple tests, the Bonferroni correction was applied to test each individual hypothesis at the level of 0.002. All analyses were performed using STATA 13.

## 3 Results

The 1899 participants enrolled had a mean age of 32.8 years at baseline, and were predominantly Hindu, employed and with at least some formal education (Table 1). Almost 60% lived in rural areas, were married or co-habiting, and belonged to the middle and highest strata of the SLI. The prevalence of AUD at baseline was 17.1% (95% CI 15.4–18.8). This included 12.4% (95% CI 11.0–14.0) hazardous drinkers and 4.6% (95% CI 3.7–5.7%) harmful drinkers.

Over the 6 year follow-up period, the proportion LTFU was 20.3%, and was over twice as high in the urban areas compared to rural areas (29.3% vs 13.4%, p < 0.001; Table 2). Other univariable predictors of LTFU were Christian religion, higher education, unemployment, and higher SLI (Table 2). In multivariable analysis, the only variable significantly associated with LTFU was living in urban areas (OR 2.8; 95%CI 2.2–3.6; p < 0.001). Notably, having AUD at baseline was not associated with LTFU (18.6% vs 20.6% among those with and without AUD respectively; Table 2). Overall, 62 participants (3.3%; 95%CI 2.5–4.2) had died at follow-up, with causes of death as follows: liver disease (17.7%), suicide (14.5%), various types of cancer (11.3%), myocardial infarct (11.3%), tuberculosis (8.1%), accidents and injuries (6.5%), other causes (renal failure, AIDS, multi-organ failure) (8.1%), and unknown cause (22.6%). The most common causes of death in those having AUD at baseline were liver disease (28%), accident and injuries (12%), and suicide (12%). After adjusting for socio-demographic factors, compared to those who did not have AUD at baseline, those with AUD had significantly higher odds of dying at follow up (OR 2.9; 95% CI 1.7–5.0).

**Fig. 1:**
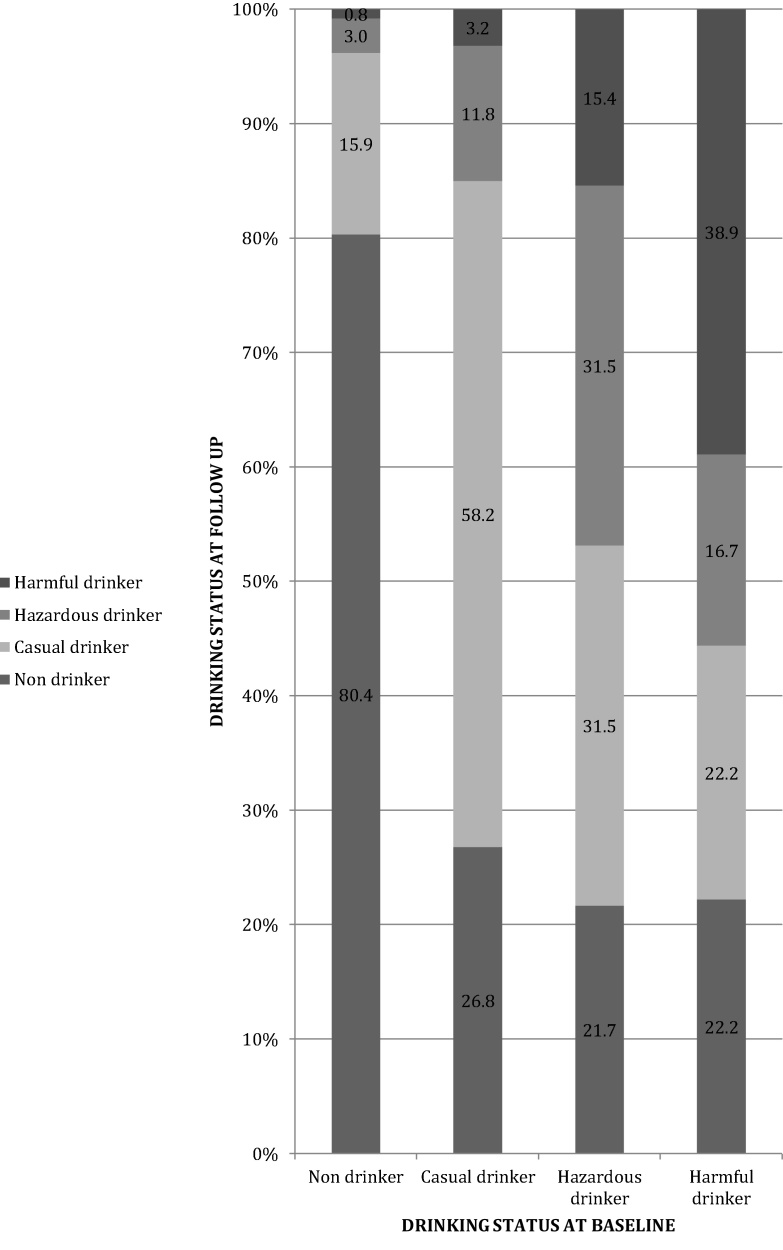
Longitudinal progression of AUD.

Fig. 1 describes how AUD status at follow-up by baseline status. Of the non-drinkers at baseline, 3.7% had AUD at follow-up, compared with 15.0% of baseline casual drinkers. Prevalence of AUD at follow-up was much higher among those with AUD at baseline (46.9% among hazardous drinkers and 55.4% among harmful drinkers). One in five (21.8%) of men with AUD at baseline had stopped drinking at follow up.

We conducted sensitivity analyses considering two potential scenarios viz all those LTFU had no AUD and all those LTFU had AUD. If all those LTFU had AUD at follow up then of the non-drinkers at baseline, 26% would have AUD at follow-up, compared with 39.4% of baseline casual drinkers. Furthermore, prevalence of AUD at follow-up would be much higher among those with AUD at baseline (61.4% among hazardous drinkers and 70.4% among harmful drinkers). If none of those LTFU had AUD at follow up then of the non-drinkers at baseline, 3.7% would have AUD at follow-up, compared with 14.5% of baseline casual drinkers. Furthermore, prevalence of AUD at follow-up would be much higher among those with AUD at baseline (35.6% among hazardous drinkers and 42.1% among harmful drinkers).

Table 3 describes the follow-up outcomes of AUD at baseline. On multivariable analysis, compared to being abstinent, casual drinking at baseline was strongly associated with tobacco use and raised GGT (p < 0.002) at follow up. Similarly, compared to being abstinent, hazardous drinking at baseline was strongly associated with tobacco use and, raised GGT and MCV (p < 0.002) at follow up. Harmful drinking at baseline was strongly associated with several factors, including workplace problems, lost workdays, social problems, hypertension, death, tobacco use, suicidality, anxiety disorders, and raised MCV and GGT (p < 0.002) at follow up. The test of trend was positive for all of these except anxiety disorders and raised GGT.

A subgroup analysis was conducted in current drinkers only. On multivariable analysis, compared to casual drinking, hazardous drinking at baseline was strongly associated with tobacco use, and raised MCV and GGT (p < 0.002) at follow up. Compared to casual drinking, harmful drinking at baseline was strongly associated with workplace problems, social problems, death, tobacco use, suicidality, and anxiety disorders (p < 0.002) follow up.

## 6 Discussion

In this unique population based long-term cohort study of AUD in men in India we examined the longitudinal course and impact of AUD in a large sample of men in Goa. We observed that a substantial number of non-drinkers (3.7%) and casual drinkers (15.0%) developed AUD over a relatively short period of six years. Furthermore half of the men who already have AUD continued to have AUD and about 1 in 6 men with less severe AUD (hazardous drinking) developed more severe AUD (harmful drinking). Conversely, over the six-year period almost a third of men with AUD become casual drinkers and almost a fifth of hazardous drinkers and harmful drinkers had stopped drinking over a six-year period. This is an especially important finding in a context where formal help for AUD is minimal. Finally, AUD at baseline was found to be associated with adverse outcomes at follow up in various domains of the drinkers’ life including social problems and interpersonal problems (e.g., workplace problems, marital problems, and perpetration of domestic violence), and physical and mental health problems (e.g., accidents, injuries, death, suicidality).

Few studies have examined the longitudinal history and impact of AUD in India (Kar et al., 2003, Kuruvilla and Jacob, 2007, Kuruvilla et al., 2004, Mohan et al., 2002a, Mohan et al., 2002b, Singh et al., 2008). All but one (Mohan et al., 2002b) were conducted among men attending clinics, and are prone to selection bias due to low help-seeking behaviours of people with AUD. The only population-based longitudinal study examining AUD in India (Mohan et al., 2002b) had an exclusively urban sample, only described the incidence of AUD but not long term outcomes of those already having AUD and had a follow-up period of only one year. Hence, one of the main strengths of our study is in terms of filling a policy relevant knowledge gap on the long-term consequences of men with AUD in a population sample.

We could not find any similar studies from other LMICs, but there are several population cohorts in developed countries with variable findings with regard to longitudinal progression of AUD. In a longitudinal study from Sweden, 48% of the surviving ‘alcoholics’ and 61% of the sample were still problem drinkers at 25 years of follow up (Ojesjo, 2000). In a national study of adults in the USA 18% of baseline frequent heavy episodic drinkers continued to be heavy episodic drinkers at the 25 year follow-up (Sloan et al., 2011). In a cohort analysis of samples of two longitudinal studies from the USA, at follow up, 62% of the older age cohort and 19% of the younger age cohort persist drinking at the same levels as at baseline (Fillmore, 1987). Finally in a cohort of ‘alcoholic’ participants, 19% participants abstained in the first year whereas 10% abstained at 3 years (Imber et al., 1976). Our findings are consistent with findings from other population cohorts from developed countries which have also demonstrated that AUD, in comparison with non-drinkers as well as casual drinkers, increases the risk for various adverse bio-psycho-social outcomes like relationship problems, social problems, domestic violence, workplace problems, accidents and injuries, and mortality (Callaghan et al., 2013, Fergusson et al., 2013, Morandi et al., 2015, Moure-Rodriguez et al., 2014, Ojesjo, 1981). Furthermore, for almost all these outcomes there appears to be a dose response relationship with increased risk of the outcomes with increasing severity of AUD (Corrao et al., 1999).

In this study we observed several adverse bio-psycho-social impacts of AUD which are statistically significant at the conventional cut off value of p = 0.05. Since we have used the Bonferroni correction to offset the influence of multiple hypotheses testing we have not considered these as statistically significant. However, it would be remiss to ignore them completely. These include marital problems, physical abuse, diabetes, COPD, accidents and injuries, and major depression. Evidence for almost all of these have been demonstrated in various studies across the developed world (Dikmen et al., 1995, Hu, 2011, Jones et al., 1995, Puddey et al., 1997, Regier et al., 1990). Furthermore, although cross sectional studies from India have demonstrated such associations, our study enhances that evidence by demonstrating associations at the conventional level of significance.

Our study has some limitations as outlined below. Although we describe the longitudinal evolution of AUD in a LMIC it is by no means a natural history of AUD because some of these participants might have received treatment for their AUD which might have influenced the course of the disorder. Although we did not collect data on health service utilisation specific to AUD treatment, it is unlikely that many participants would have received such treatment as access to care for AUD is very low (Kohn et al., 2004). Another limitation is that, due to the fluctuating course of AUD, someone who had AUD at baseline and follow up might have had an extended period of abstinence in the intervening period but would be erroneously labelled as having persistent AUD. Similarly a participant drinking casually at baseline and follow up, might have been drinking harmfully in the intervening period which would not be captured by our study design. Thus, we can only conclude from these data the association between the exposure at baseline and current AUD status. Furthermore, since we did not have baseline data of the various outcomes measured at follow up, we could not adjust for those at baseline. This in turn means that we can make conclusions regarding associations (and not causality) between baseline AUD and adverse outcomes at follow up. Urban residence at baseline was significantly associated with LTFU. Possible reasons for this include the itinerant and seasonal nature of the population in the touristy areas and the rapid and poorly planned urbanisation making it difficult to trace the addresses collected in the baseline survey. As urbanicity is associated with higher rates of substance use disorders, it is possible that differential LTFU could have led to an under-estimate of the prevalence of AUD at follow up in urban areas. However, the higher LTFU would not bias the association of baseline AUD with the range of adverse bio-psycho-social outcomes at follow-up as we adjusted for area of residence while testing those associations. Finally many measurements in our study, including alcohol use, are self-reported and social desirability is bound to affect participant responses. However, there is evidence to suggest that, given adequate privacy and confidence about confidentiality, research participants give reliable and accurate information even about sensitive information like substance use (Darke, 1998). Furthermore, we also collected data on biological parameters to supplement the self-reported data. Besides being the largest long-term population based study of AUD in an Indian setting, our study has several strengths including: measurement of multiple exposures and outcomes, absence of non random misclassification of exposure status, and reduction of non random misclassification of outcomes based on exposure status by blinding the research workers to the exposure status.

India is a heterogeneous culture and as cultural context is an important determinant of alcohol use the uniform generalisability of our findings across the country has to be treated with caution. Despite this, our findings have several clinical, research and policy implications. One major finding is that half of all AUD remits even within a context where services for AUD are minimal. Furthermore drinking status as well as AUD status changes greatly over relatively short periods of time in these settings. A key research priority is to examine the predictors of such changes, i.e., development of new AUD, persistence of existing AUD, and recovery as these will inform the priorities for programmes for the prevention and treatment of AUD. Data from this cohort will be separately analysed to examine such predictors of various trajectories of AUD. Policymakers too need to take into consideration the high rate of conversion of casual drinking to AUD and the long term impact of drinking on a range of domains of the drinker’s life and accordingly plan integrated alcohol policies which target the problem at various levels, e.g., drunk driving penalties, taxation, and development of relevant health services. Research also needs to examine the mechanisms leading to the various negative long term outcomes of drinking, and the interactions between them, as this will allow the development of complex interventions which can target the disorder at various levels. Interestingly, although there is increasing risk of adverse health outcomes with increasing severity of AUD, there is no concomitant increase in health service utilisation, indicating the need for more demand side interventions. These could include implementing routine screening and brief interventions delivered by non-specialist health workers integrated into existing healthcare platforms, e.g., primary care as demonstrated in a treatment development project in Goa (Nadkarni et al., 2015). Furthermore, a key finding of dose response relationship for most of these associations warrants further investigation of the causal relationship between AUD and the outcomes studied. Finally, our findings show the universality of the longitudinal course and outcome of AUD across very different contexts. This could mean that policies, services and interventions developed in other contexts could have relevance to Indian settings.

To conclude, substantial numbers of non-drinkers/casual drinkers develop and have persistent AUD over a relatively short period of time; and suffer long term adverse impact on various domains of their lives. This is an important addition to the literature on the course and outcome of AUD in LMIC and can be an important driver to influence health policy in such settings.

## Conflict of interest

No conflict declared.

## Funding

This work was supported by the Wellcome Trust Research Training Fellowship to Abhijit Nadkarni [grant number WT093897MA]. Vikram Patel is supported by a Wellcome Trust Senior Research Fellowship.The funding agency had no role in the analysis and interpretation of data; in the writing of the manuscript; or in the decision to submit the paper for publication.

## Contributors

Abhijit Nadkarni led the cohort study, conceived the paper, did the analyses, and wrote up the findings. Helen Weiss supported in the drafting of the analyses plan and commented on the first draft of the manuscript and subsequent revisions. Vikram Patel supervised Abhijit in conducting the cohort study, supported in conceiving the paper, and commented on the first draft of the manuscript and subsequent revisions. Aresh Naik and Bhargav Bhat coordinated the cohort study in the field, supervised the data collection, checked the quality of the data, and commented on the first draft of the manuscript and subsequent revisions. All authors read and approved the final manuscript.

## Figures and Tables

**Table 1:** Baseline socio-demographic profile of the cohort.

Variable	Abstainer at baseline Mean (SD) or n (%) n = 1133 (59.7%)	Casual drinker at baseline Mean (SD) or n (%) n = 442 (23.3%)	Hazardous drinker at baseline Mean (SD) or n (%) n = 236 (12.4%)	Harmful drinker at baseline Mean (SD) or n (%) n = 88 (4.6%)	p value
Mean age in years (SD)*	32.3 (9.0)	32.8 (8.4)	34.3 (8.0)	35.2 (7.5)	

Residence					
Rural	721 (67.0)	195 (18.1)	108 (10.0)	53 (4.9)	<0.001
Urban	412 (50.1)	247 (30.1)	128 (15.6)	35 (4.3)	

Religion					
Hindu	998 (61.8)	356 (22.0)	187 (11.6)	75 (4.6)	<0.001
Muslim	63 (64.3)	16 (16.3)	14 (14.3)	5 (5.1)	
Christian	71 (38.6)	70 (38.0)	35 (19.0)	8 (4.4)	

Marital status					
Married or co-habiting	617 (57.3)	250 (23.2)	152 (14.1)	58 (5.4)	0.01
Never married/divorced/separated/widowed	516 (62.8)	192 (23.4)	84 (10.2)	30 (3.7)	

Education					
No formal education	58 (62.4)	13 (14.0)	12 (12.9)	10 (10.8)	<0.001
Completed primary	79 (57.3)	23 (16.7)	22 (15.9)	14 (10.1)	
Completed secondary	578 (56.8)	236 (23.2)	148 (14.6)	55 (5.4)	
Completed higher secondary	188 (59.1)	87 (27.4)	35 (11.0)	8 (2.5)	
Graduate and above	204 (71.1)	65 (22.7)	17 (5.9)	1 (0.4)	

Employment status					
Employed	971 (58.2)	403 (24.2)	215 (12.9)	79 (4.7)	0.007
Unemployed	162 (70.1)	39 (16.9)	21 (9.1)	9 (3.9)	

Standard of Living Index					
Lowest 40% (Poor)	443 (61.5)	130 (18.1)	92 (12.8)	55 (7.6)	<0.001
Middle 40%	443 (58.2)	190 (25.5)	96 (12.9)	25 (3.4)	
Highest 20% (Rich)	255 (58.9)	122 (28.2)	48 (11.1)	8 (1.9)	

* Only significant differences are hazardous drinkers vs abstainers (p = 0.006) and harmful drinkers vs abstainers (p = 0.018).

**Table 2:** Predictors of dropout from the cohort.

Variable	Follow up data available n = 1514 (79.7%) n (%)	Dropouts n = 385 (20.3%) n (%)	p value
Mean age in years (SD)	33.4 (8.4)	32.4 (8.7)	0.12

Residence			
Rural	933 (86.6)	144 (13.4)	<0.001
Urban	581 (70.7)	241 (29.3)	

Religion			
Hindu	1325 (82.0)	291 (18.0)	<0.001
Muslim	72 (73.5)	26 (26.5)	
Christian	117 (63.6)	67 (36.4)	

Marital status			
Married or co-habiting	873 (81.1)	204 (18.9)	0.09
Never married/divorced/separated/widowed	641 (78.0)	181 (22.0)	

Education			
No formal education	74 (79.6)	19 (20.4)	<0.001
Completed primary	124 (89.9)	14 (10.1)	
Completed secondary	834 (82.0)	183 (18.0)	
Completed higher secondary	237 (74.5)	81 (25.5)	
Graduate and above	207 (72.1)	80 (27.9)	

Employment status			
Unemployed	172 (74.5)	59 (25.5)	0.03
Employed	1342 (80.5)	326 (19.5)	

Standard of Living Index			
Lowest 40% (Poor)	607 (84.3)	113 (15.7)	0.001
Middle 40%	575 (77.3)	169 (22.7)	
Highest 20% (Rich)	331 (76.4)	102 (23.6)	

AUD			
No	1295 (79.5)	335 (20.6)	0.46
Yes	219 (81.4)	50 (18.6)	

**Table 3:** Longitudinal impact of AUD.

	Univariate analyses (Comparator group is abstainers)			Multivariate analyses (Comparator group is abstainers)*		
	Casual drinkers OR (95% CI), p	Hazardous drinkers OR (95% CI)	Harmful drinkers OR (95% CI)	Casual drinkers OR (95% CI)	Hazardous drinkers OR (95% CI)	Harmful drinkers OR (95% CI)
Social problems						
Workplace problems since baseline interview	1.3 (0.6–2.8)	2.5 (1.1–5.5)†	7.2 (3.1–17.1)†	1.7 (0.7–3.7)	3.0 (1.3–7.0)††	7.1 (2.7–18.2)^3^
Marital problems since baseline interview	0.8 (0.5–1.5)	2.1 (1.2–3.7)†	3.3 (1.6–7.1)†	0.9 (0.5–1.7)	2.0 (1.1–3.7)††	2.9 (1.3–6.4)^2^
Social problems since baseline interview	0.8 (0.4–1.9)	2.4 (1.1–5.2)†	5.5 (2.2–13.4)†	0.9 (0.4–2.3)	2.6 (1.1–6.0)††	5.2 (2.0–13.7)^3^
Lost ≥ 1 workdays due to poor health in past 28 days	1.1 (0.8–1.7)	1.1 (0.7–1.9)	3.3 (1.7–6.4)†	1.3 (0.9–1.9)	1.2 (0.7–2.0)	3.3 (1.7–6.5)^3^
Physical abuse of partner/spouse in past 12 months	0.9 (0.4–2.1)	1.9 (0.9–4.3)	4.2 (1.6–11.0)†	1.1 (0.5–2.4)	2.1 (0.9–4.8)	3.8 (1.4–10.1)^2^
Sexual abuse of partner/spouse in past 12 months	0.7 (0.5–1.0)†	0.7 (0.5–1.0)†	0.9 (0.5–1.7)	0.8 (0.5–1.1)	1.0 (0.6–1.7)	1.5 (0.7–3.2)

Physical health problems						
Hypertension diagnosed after baseline interview	1.0 (0.7–1.5)	1.4 (0.9–2.2)	2.7 (1.5–4.9)†	1.0 (0.7–1.6)	1.3 (0.8–2.2)	3.0 (1.6–5.6)^3^
Heart disease diagnosed after baseline interview	0.8 (0.3–2.1)	1.1 (0.4–3.2)	1.5 (0.3–6.6)			
CVA or TIA occurring after baseline interview	0.9 (0.3–2.2)	0.6 (0.1–2.4)	1.6 (0.4–7.0)			
Head injury with loss of consciousness after baseline interview	1.0 (0.6–1.8)	1.8 (1.0–3.2)†	1.5 (0.6–3.8)	1.2 (0.7–2.1)	1.8 (1.0–3.3)	1.3 (0.5–3.5)
Diabetes diagnosed after baseline interview	1.7 (1.0–2.6)†	2.1 (1.2–3.6)†	2.3 (1.0–5.1)†	1.8 (1.1–2.9)††	2.2 (1.3–4.0)††	3.0 (1.3–6.8)††
COPD diagnosed after baseline interview	1.2 (0.5–3.3)	1.6 (0.5–4.9)	5.8 (2.0–16.8)†	1.7 (0.6–4.6)	2.0 (0.6–6.3)	5.2 (1.7–16.1)††
Tuberculosis diagnosed after baseline interview	0.2 (0.1–1.1)†	1.2 (0.4–3.2)	2.1 (0.6–7.1)	0.2 (0.02–1.2)	1.2 (0.4–3.4)	1.6 (0.4–5.7)
Accidents or injuries in past 12 months	1.0 (0.7–1.5)	1.8 (1.2–2.8)†	2.6 (1.4–4.8)†	1.1 (0.7–1.6)	1.8 (1.2–2.9)††	2.5 (1.3–4.6)††
Death	1.2 (0.6–2.5)	2.0 (0.9–4.1)†	9.1 (4.6–18.0)†	1.5 (0.7–3.0)	1.8 (0.8–3.9)	6.2 (3.0–12.5)†††

Mental health and substance use/abuse						
Used tobacco in past 12 months	1.4 (1.1–1.8)†	3.5 (2.6–4.9)†	4.2 (2.6–6.7)†	1.9 (1.4–2.6)†††	4.2 (2.9–6.0)†††	3.4 (2.0–5.6)†††
Current major depressive episode	0.3 (0.1–1.0)†	1.0 (0.4–2.4)	3.4 (1.5–8.2)†	0.4 (0.1–1.2)	1.2 (0.5–2.9)	3.1 (1.2–7.6)††
Currently suicidal	0.9 (0.6–1.5)	1.5 (0.8–2.5)	4.7 (2.5–8.6)†	1.2 (0.7–2.0)	1.7 (1.0–3.0)	4.2 (2.2–7.9)†††
Current anxiety disorders	0.4 (0.2–1.2)†	0.7 (0.2–1.9)	4.7 (2.1–10.4)†	0.6 (0.2–1.6)	0.8 (0.3–2.3)	4.2 (1.8–9.6)†††
Current substance use disorder	2.7 (0.4–19.2)	7.7 (1.3–46.7)†	7.1 (0.6–79.6)	1.6 (0.2–11.3)	4.9 (0.8–30.7)	6.2 (0.5–78.9)

Health service utilisation						
Contact with health worker in past 2 months	1.1 (0.8–1.4)	0.8 (0.6–1.2)	1.3 (0.8–2.1)			
Admitted to hospital in the past two months	1.3 (0.5–3.0)	1.8 (0.7–4.7)	3.5 (1.1–10.6)†	1.4 (0.6–3.5)	1.8 (0.7–4.8)	2.8 (0.9–8.8)

Biological parameters						
Hypertension	1.5 (1.0–2.4)†	1.3 (0.7–2.4)	1.8 (0.8–4.2)	1.2 (0.8–2.0)	1.1 (0.6–2.1)	1.8 (0.7–4.1)
Unhealthy BMI	1.1 (0.9–1.5)	1.3 (0.9–1.8)	1.1 (0.7–1.9)			
Raised MCV	1.3 (1.0–1.8)†	3.1 (2.2–4.5)†	3.5 (2.0–6.2)†	1.3 (1.0–1.8)	2.8 (1.9–4.2)†††	3.1 (1.7–5.5)†††
Raised GGT	3.2 (2.1–4.7)†	7.9 (5.1–12.2)†	8.6 (4.7–15.9)†	3.7 (2.5–5.5)†††	8.7 (5.5–13.6)†††	8.9 (4.7–16.8)†††

* Adjusted for age, residence, religion, marital status, education, employment status and socioeconomic status at baseline.

† Significance level of p < 0.1; progressed to the multivariate model.

†† Significance level of p < 0.05 but >0.002.

††† Significance level of p < 0.002.
