# Supplementary material for: The six-year outcome of alcohol use disorders in men: A population based study from India
Source: Drug Alcohol Depend. 2016 May 1;162:107–15. doi: 10.1016/j.drugalcdep.2016.02.039 (PMC4841788; doi:10.1016/j.drugalcdep.2016.02.039)
Supplement: Supplementary file 1 [file mmc1.docx]

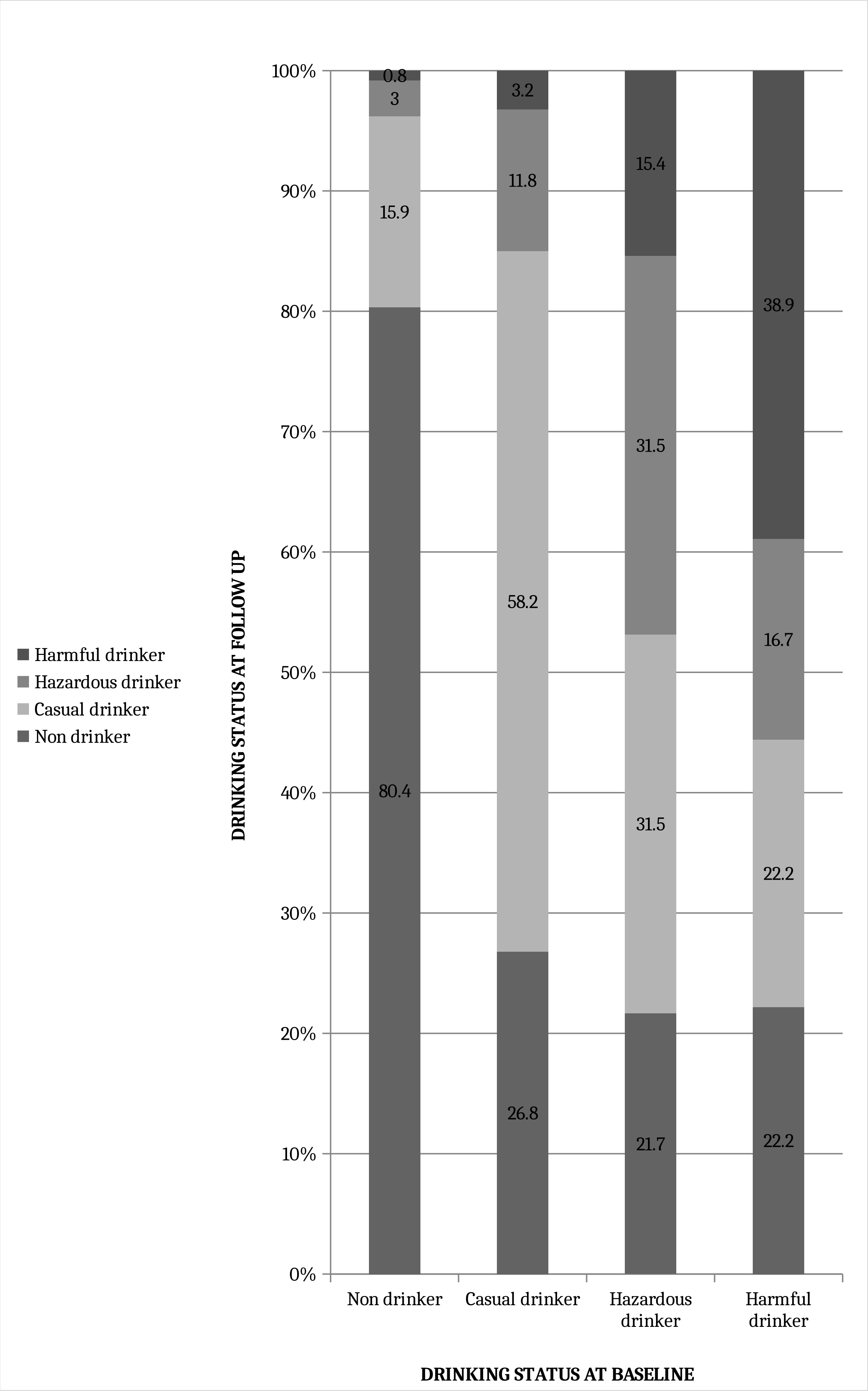
Harmful drinker
Hazardous drinker
Casual drinker
Non drinker
DRINKING STATUS AT FOLLOW UP
100%
90%
80%
70%
60%
50%
40%
30%
20%
10%
0%
0.8
3
15.9
80.4
3.2
11.8
58.2
26.8
15.4
31.5
31.5
21.7
38.9
16.7
22.2
22.2
Non drinker
Casual drinker
Hazardous drinker
Harmful drinker
DRINKING STATUS AT BASELINE
